# High-Entropy Prussian Blue Analogue Derived Heterostructure Nanoparticles as Bifunctional Oxygen Conversion Electrocatalysts for the Rechargeable Zinc–Air Battery

**DOI:** 10.1021/acsami.4c13387

**Published:** 2024-11-04

**Authors:** Wuttichai Tanmathusorachai, Sofiannisa Aulia, Mia Rinawati, Ling-Yu Chang, Chia-Yu Chang, Wei-Hsiang Huang, Ming-Hsien Lin, Wei-Nien Su, Brian Yuliarto, Min-Hsin Yeh

**Affiliations:** † Department of Chemical Engineering, National Taiwan University of Science and Technology, Taipei 10607, Taiwan; ‡ Department of Chemical Engineering and Biotechnology, National Taipei University of Technology, Taipei 10608, Taiwan; § Graduate Institute of Applied Science and Technology, National Taiwan University of Science and Technology, Taipei 10607, Taiwan; ∥ National Synchrotron Radiation Research Center, Hsinchu 30076, Taiwan; ⊥ Department of Chemical and Materials Engineering, Chung Cheng Institute of Technology, National Defense University, Dasi, Taoyuan 335, Taiwan; # Sustainable Electrochemical Energy Development Center, National Taiwan University of Science and Technology, Taipei 10607, Taiwan; % Advanced Functional Materials Laboratory, Department of Engineering Physics, Institute of Technology Bandung (ITB), Bandung 40132, Indonesia

**Keywords:** Prussian blue analogues, high entropy, heterostructure nanoparticles, bifunctional electrocatalyst, rechargeable zinc−air battery

## Abstract

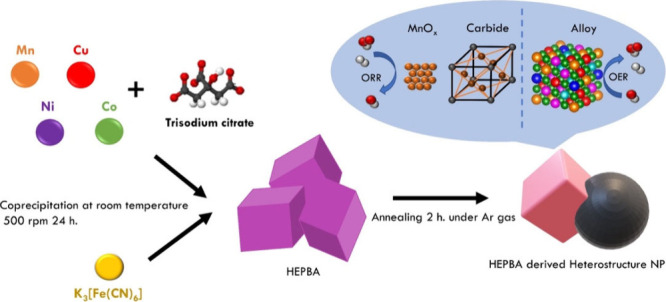

In response to energy challenges, rechargeable zinc–air batteries (RZABs) serve as an ideal platform for energy storage with a high energy density and safety. Nevertheless, addressing the sluggish oxygen reduction reaction (ORR) and oxygen evolution reaction (OER) in RZAB requires highly active and robust electrocatalysts. High-entropy Prussian blue analogues (HEPBAs), formed by mixing diverse metals within a single lattice, exhibit enhanced stability due to their increased mixing entropy, which lowers the Gibbs free energy. HEPBAs innately enable sacrificial templating, an effective way to synthesize complex structures. Impressively, in this study, we successfully transform HEPBAs into exquisite multiphase (multimetallic alloy, metal carbide, and metal oxide) heterostructure nanoparticles through a controlled synthesis process. The elusive multiphase heterostructure nanoparticles manifested two active sites for selective ORR and OER. By integrating CNT into HEPBA-derived nanoparticles (HEPBA/CNT-800), the HEPBA/CNT-800 demonstrates superior activity toward both ORR (*E*_1/2_ = 0.77 V) in a 0.1 M KOH solution and the OER (η = 330 mV at 50 mA cm^–2^) in a 1 M KOH solution. The RZAB with a HEPBA/CNT-based air electrode demonstrated an open-circuit voltage of 1.39 V and provided a significant energy density of 71 mW cm^–2^. Moreover, the charge and discharge cycles lasting up to 40 h at a current density of 5 mA cm^–2^ demonstrate its excellent stability. This work provides an alternative avenue for the rational design of HEPBA’s derivative for a sustainable rechargeable metal–air battery platform.

## 1 Introduction

Significant efforts have focused on developing sustainable energy storage, conversion, and ecofriendly technologies in response to growing energy and environmental challenges. Metal–air batteries (MABs) demonstrate excellent and promising candidates in this pursuit, with rechargeable zinc–air batteries (RZABs) particularly notable due to their high energy density, cost-effectiveness, and safety.^1−6^ However, the RZABs’ performance is restricted by the sluggish cathode and kinetics of the oxygen reduction reaction (ORR, 4-electron transfer pathway reactions) and the oxygen evolution reaction (OER). Traditional precious-metal electrocatalysts such as Pt, IrO_2_, and RuO_2_ demonstrate superior activity as benchmarks for the OER and ORR, but they are constrained by their high price, insufficiency, and stability.^6−10^ As a consequence, the investigation into bifunctional electrocatalysts in the field of nonprecious-metal-based materials continues as an alternative.

High-entropy (HE) concepts have attracted growing interest in recent years and accelerated the development of many multicomponent (equimolar) materials for a wide range of applications. High-entropy materials are single-phase materials containing five or more metallic elements in similar atomic proportions, believed to be stabilized by high configurational entropy, which promotes chemical and structural diversity. High-entropy Prussian blue analogues (HEPBAs) are materials that have not yet raised any concerns in the metal–air battery electrode materials field.^11^ Many types of metal–organic frameworks (MOFs) that have been studied for electrocatalysts are known to possess high stability due to the unique framework and fairly distributed metal nodes. One of the subclasses of MOFs is the Prussian blue analogues (PBAs), which are easy to synthesize, nontoxic, and affordable. The beneficial properties of PBAs make them suitable for various applications such as sensors, electrocatalysis, photocatalysis, and zinc–air batteries.^12,13^ However, their limited durability, surface area, and poor electrical conductivity hinder their widespread use in oxygen electrocatalysis. Additionally, PBAs are often changed into carbides, metallic alloys, oxides, hydroxides, sulfides, or phosphides as part of plans to enhance activity.^14−16^

Moreover, high-entropy Prussian blue analogues hold promise for developing excellent cathode components for rechargeable zinc–air batteries (RZABs), owing to their high tunability and versatility.^17^ A variety of high-entropy (HE) material derivatives, which include initial high-entropy alloys (HEAs), high-entropy carbides (HECs), high-entropy oxides (HEOs), high-entropy oxyfluorides (HEOFs), and high-entropy MXenes (HEMXs), have found applications in electrochemical energy storage catalysis and are environmentally friendly, showcasing the significant enhancement in cyclic performance achievable by incorporating high-entropy principles into battery materials.^11,18−22^ The high-entropy concept has been remarkably applied to transition metal carbide systems. Because of their higher catalytic activity, high electrical conductivity, corrosion resistance, and platinum-like behavior in chemisorbing hydrogen and oxygen, researchers have also investigated transition-metal carbides (TMCs) for catalytic applications.^23,24^ 3D crystalline high-entropy transition-metal carbides exhibit properties that outperform those of mono-transition-metal carbides, including low thermal conductivity, improved hardness, and oxidation resistance,^25^ leading to their utilization in thermal, corrosive, and extreme temperature and pressure environments.

**Scheme 1:**
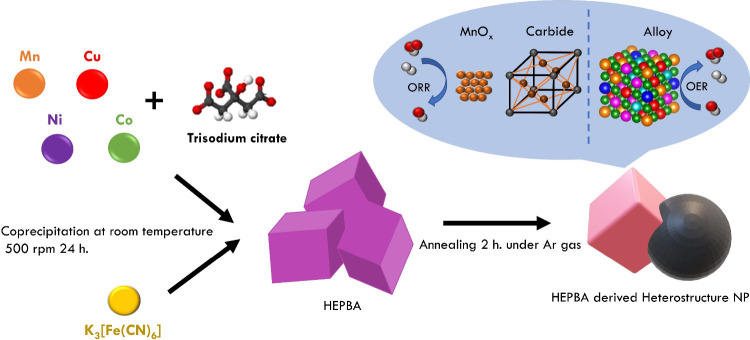
Synthetic Strategy for HEPBA-Derived Heterostructure Nanoparticle

In this study, we report on successfully synthesizing HEPBAs through a simple coprecipitation method. Once we confirmed the unique properties owned by HEPBAs, we used a pyrolysis treatment (HEPBA-*T*) at different temperatures to control the change from HEPBAs to transition-metal oxide, carbide, and alloy phases (Scheme 1). Optimizing the proportion of phase contents can not only effectively improve the electrocatalytic performance for both ORR and OER but also decrease the charge-transfer resistance. The as-synthesized HEPBA-800 demonstrates exceptional ORR and OER performance owing to its specific structure and chemical composition. This finding indicates a promising bifunctional electrocatalyst in the air cathode.

## 2 Experimental Section

### 2.1 Materials

The materials were employed in their original state without any further purification, depending solely on their initial quality and requirements. Potassium hexacyanoferrate (K_3_(Fe(CN)_6_, ≥99.0%) and cobalt(II) nitrate hexahydrate (Co(NO_3_)_2_·6H_2_O, ≥99%) were purchased from J.T.Baker. Nickel nitrate hexahydrate (Ni(NO_3_)_2_·6H_2_O, ≥98%) and ruthenium(IV) oxide hydrate (99.99% purity) were obtained from Alfa Aesar. Copper(II) chloride dihydrate (CuCl_2_·2H_2_O) was obtained from Choneye with a purity of ≥98%. Sodium citrate tribasic dihydrate (HOC(COONa)-(CH_2_COONa)_2_·2H_2_O, ≥99%), manganese(II) sulfate monohydrate (MnSO_4_·H_2_O, ≥98%), isopropanol, a Nafion 117 solution (5% in a mixture), and Pt/C (20% Pt on Vulcan XC72) were provided by Sigma-Aldrich. UniRegion Bio-Tech provided a multiwalled carbon nanotube with an average length of approximately between 5 and 15 μm and an outer diameter of less than 10 nm. We purchased nitric acid (HNO_3_, 65%) from PanReac AppliChem to functionalize the CNT. Deionized water (DIW) was utilized in the experiment with a specific resistance of 18.2 MΩ cm^–1^ at 25 °C. Potassium hydroxide (KOH) was sourced from Fisher Chemical.

### 2.2 Preparation of HEPBA and Its Derivations

High-entropy Prussian blue analogue (HEPBA) was synthesized by using a simple coprecipitation process at room temperature with water as the solvent, according to the literature. Briefly, Co(NO_3_)_2_·6H_2_O, Ni(NO_3_)_2_·6H_2_O, MnSO_4_·4H_2_O, and CuCl_2_·2H_2_O with 0.5 mmol per each of Co/Ni/Mn/Cu and 2.25 mmol of sodium citrate dihydrate were dissolved and mixed in 50 mL of DI water to obtain solution A. Then, 2.5 mmol of K_3_(Fe(CN)_6_) was dissolved in 50 mL of DI water to obtain solution B. While stirring at 500 rpm, solution B was slowly dropped into solution A and continuously stirred for 24 h. We centrifuged the solution and subsequently dried it for 12 h at 70 °C. High-entropy Prussian blue analogue derived (HEPBA-800) was obtained by subjecting the synthesized material to pyrolysis treatment in a tubular furnace at 800 °C with a heating rate of 5 °C min^–1^ and maintaining that temperature for 2 h under an argon atmosphere. The samples HEPBA-700 and HEPBA-900 were also prepared using the same method, adjusting the annealing temperature at 700 and 900 °C, respectively. As a comparison to the bimetallic PBA, the NiFe PBA was synthesized with the same procedure only with Ni(NO_3_)_2_·6H_2_O and K_3_(Fe(CN)_6_) as the precursor. The composition of 0.5 mmol was used for both the Ni and Fe precursors.

### 2.3 Preparation of an Electrode with Electroactive Materials

The electrochemical measurements were conducted using a three-electrode system in an Autolab digital potentiostat/galvanostat (PGSTAT302N with FRA32M, Metrohm) at an ambient temperature of 25 °C. The Ni foam electrode substrate (FuCell Co., Ltd., Taoyuan, Taiwan) utilized for evaluating the OER performance underwent ultrasonic cleaning with 1.0 M HCl for 30 min, followed by multiple washes washed with DI water and subsequent drying on a hot plate at 70 °C. To prepare the HEPBA-T electrocatalyst ink (1 mL), 10 mg of the electrocatalysts was dissolved in a solution mixture containing 200 μL of DI water, 750 μL of isopropyl alcohol (IPA), and 50 μL of Nafion 117 solution. This mixture was ultrasonicated to achieve a uniform solution. The resulting catalyst solution was then applied to a Ni foam (area: 1 cm × 1 cm) using the drop-casting method (loading: 3.0 mg mL^–1^) and dried on a hot plate at 70 °C. The ORR performance was evaluated using a three-electrode cell, utilizing a rotating disc electrode (GC RDE) serving as the working electrode. The counter electrode employed in this study was platinum wire, while the reference electrode employed was Ag/AgCl (saturated KCl) at a concentration of 0.1 M KOH. The solution that was prepared was applied to the glassy carbon surface at a loading of 0.51 mg cm^–2^.

### 2.4 Structural Characterization

Morphological characterization was performed using a scanning electron microscope (JSM-7900F, JEOL) and a field-emission transmission electron microscope (FE-TEM, JEM-2100F, JEOL). The National Synchrotron Radiation Research Center (NSRRC) in Hsinchu, Taiwan, provided X-ray absorption spectroscopy (XAS) and X-ray photoelectron spectroscopy (XPS), which were conducted at the Taiwan Photon Source (TPS), specifically at beamlines BL20A, BL24A1, and BL17C1. The equipment provided valuable information about modifications in electron states and the intricate structural characteristics of the synthesized materials. XPS data were standardized using the adventitious carbon peak at 248.8 eV in the C 1s spectrum. The X-ray diffraction pattern was obtained with an X-ray diffractometer (D8 Advance, Bruker).

### 2.5 Calculation of the High-Entropy Material System

Generally, the entropy of a material system (Δ*S*_mix_) consists of four components: configurational entropy (Δ*S*_conf_), vibrational entropy (Δ*S*_vib_), electronic entropy (Δ*S*_elec_), and magnetic entropy (Δ*S*_mag_). The quantitative relationship between these components can be expressed as eq 1:
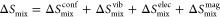
1In solid solutions, configurational entropy plays the most significant role in the overall entropy of the system. To avoid the need for complicated calculations or simulations, we simplify by using configurational entropy to represent the system’s mixing entropy, which can be calculated as eq 2:^26^
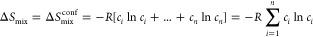
2where *R* is the gas constant, *c*_*i*_ is the mole fraction of the *i*th component, and *n* is the element’s number.

### 2.6 Electrochemical Analysis

To evaluate the oxygen evolution reaction (OER) performance, the experimental setup utilized a traditional three-electrode cell system in an oxygen-saturated environment at ambient temperature. We coated Ni foam with the designed electroactive materials, which then functioned as the working electrodes. A platinum plate (1 × 4 cm^2^) and Hg/HgO (in 1 M NaOH) served as the counter electrode and reference electrode in 1 M KOH, respectively. Linear sweep voltammetry (LSV) was carried out at a scan rate of 5 mV s^–1^, incorporating an *iR* correction to account for resistance in the electrochemical system. The ohmic drop was assessed after each measurement using impedance spectroscopy, which was subsequently employed for *iR* correction. Electrochemical impedance spectroscopy (EIS) was conducted with a 5 mV amplitude throughout a frequency range of 100 kHz to 0.1 Hz, providing a detailed understanding of the system’s behavior. To ensure consistency in reporting, all potentials were standardized to the reversible hydrogen electrode (RHE) scale using eq 3:
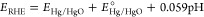
3The symbol *E*_Hg/HgO_ denotes the altered potential vs Hg/HgO, where *E*°_Hg/HgO_ equals 0.165 V at a temperature of 25 °C.

The as-prepared solution was applied to the glassy carbon surface at 0.51 mg cm^–2^ loading for the ORR performance evaluation. The linear sweep polarization curves were obtained at 5 mV s^–1^ at a rotating speed of 1600 rpm in a 0.1 M KOH solution purged with O_2_ for 30 min. The Koutecky–Levich (K–L) equation was employed to determine the electron transfer number obtained from LSV at different rotational speeds. The K–L equation is displayed below.
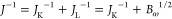
4
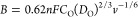
5
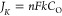
6The crucial parameters in the above-mentioned equations are delineated in this order: The observed current density was denoted as *J*, whereas *J*_L_ and *J*_K_ reflect the limiting and kinetic current densities, respectively. The Levich slope is denoted as *B*, the RDE rotation speed is represented by ω, the electron-transfer number is denoted as *n*, the Faraday constant (96485 C mol^–1^) is denoted as *F*, and the electron-transfer rate constant is denoted as *k*. The constants values for the 0.1 M KOH solution are the diffusivity of oxygen (*D*_O_ = 1.9 × 10^–5^ cm^2^ s^–1^), the electrolyte kinematic viscosity (υ = 0.01 cm^2^), and the O_2_ concentration in the solution (*C*_O_ = 1.2 × 10^–6^ mol cm^–3^).

### 2.7 Aqueous Rechargeable Zinc–Air Battery Assembly

The handmade cell was used to conduct the rechargeable zinc–air battery application. Carbon paper was utilized as the catalyst support and gas diffusion layer for oxygen transformation in the design of the cathode component. The loading was established at a constant value of 1 mg cm^–2^, while the active catalytic component possessed a square shape with an area of 4 cm^2^. The catalyst solution was generated using a methodology identical with the electrochemical performance evaluation in the three-electrode system. The electrolyte was synthesized by merging a solution of 6 M KOH with a 0.2 M solution of zinc acetate. An anode part was composed of polished Zn foil and matched in area. The battery stability and charge–discharge capacity were evaluated using a Biologic modular 6-channel electrochemical workstation over a cycle duration of 15 min, under a current density of 10 mA cm^–2^.

## 3 Results and Discussion

### 3.1 Characterization of the HEPBA

**Figure 1:**
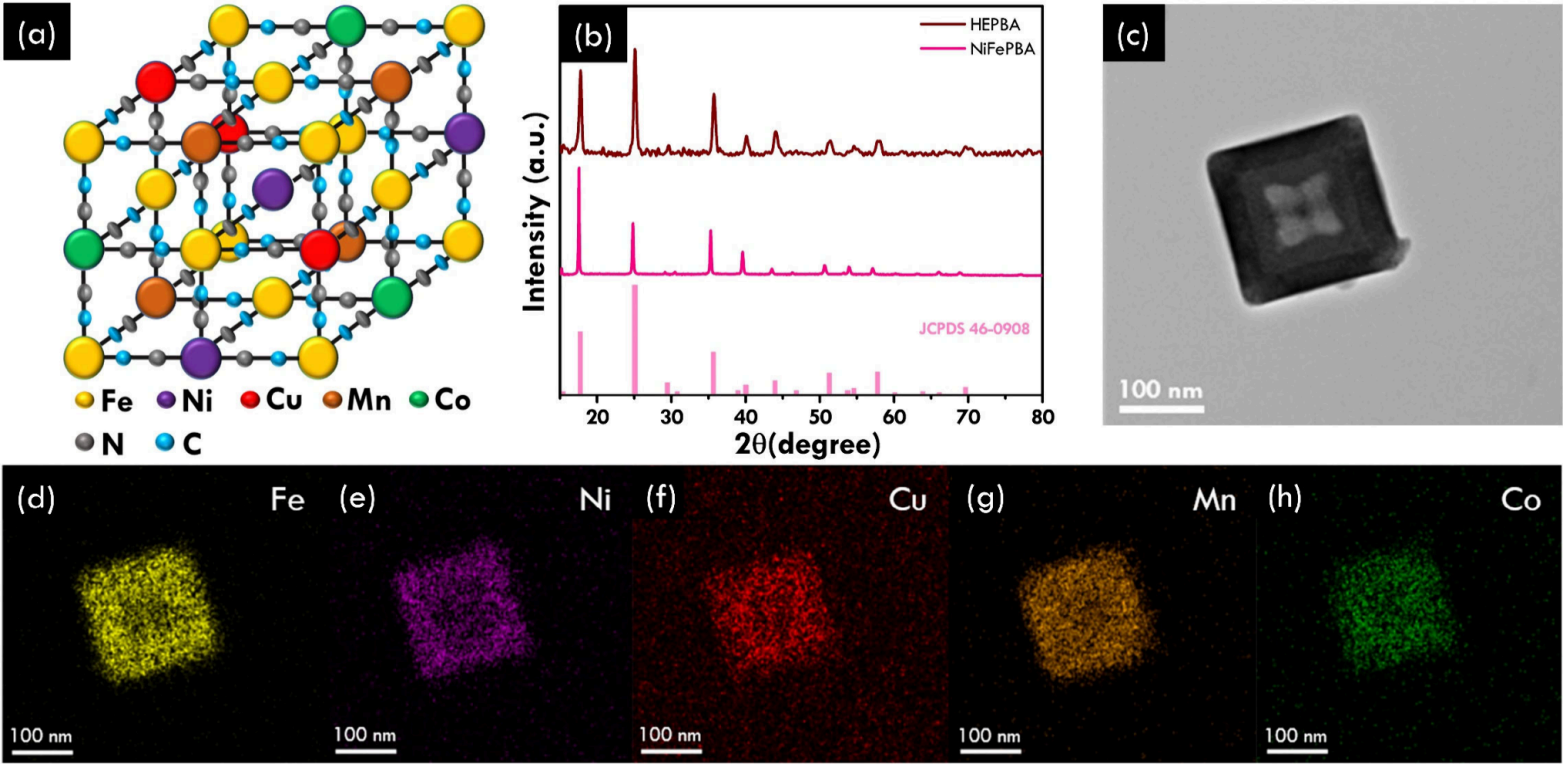
Characterization of HEPBA. (a) Illustration of the structure of HEPBA; (b) XRD pattern of HEPBA and NiFePBA; (c) TEM image of HEPBA; EDS elemental mapping images of (d) Fe, (e) Ni, (f) Cu, (g) Mn, and (h) Co for HEPBA.

The HEPBA was synthesized using nickel nitrate, cobalt nitrate, manganese sulfate, and copper chloride in 1:1:1:1 molar ratio of Co/Ni/Cu/Mn and trisodium citrate with potassium ferricyanide by a simple coprecipitation method at room temperature in the water solvent. The HEPBA powder was acquired through multiple washes with deionized water, then centrifugation, and overnight drying in an oven fixed at a temperature of 70 °C. Figure 1a illustrates the structural composition of HEPBA. The as-synthesized HEPBA possesses a cubical structure with the average size of 200–300 nm (Figure S1). The morphologies were further characterized by the X-ray diffraction pattern (XRD) and transmission electron microscopy (TEM). XRD analysis confirmed the as-synthesized HEPBA and NiFe-PBA, as shown in Figure 1b. All of the diffraction peaks are quite matched to the face-centered cubic phase of Ni_2_[Fe(CN)_6_] (JCPDS card no. 46-0908), suggesting that the two PBA samples were successfully synthesized and possessed excellent crystallinity without any additional impurities detected. Furthermore, the XRD patterns of NiFePBA and HEPBA reveal that the (200) peaks of PBA are observed at 17.5° and 17.8°, respectively. The shift suggests that introducing multimetals leads to a certain degree of lattice contraction compared to binary PBAs.^11^ The TEM image (Figure 1c) displayed an identical morphology of HEPBA, exhibiting a regular cubic shape with a particle size of approximately 180 nm, with no evidence of structural alterations. EDS elemental mapping images (Figure 1d–h) also demonstrated a uniform distribution of Mn, Fe, Co, Ni, and Cu within the PBA framework, confirming the successful synthesis of HEPBA.

### 3.2 Characterization of the Annealed HEPBA Material

**Figure 2:**
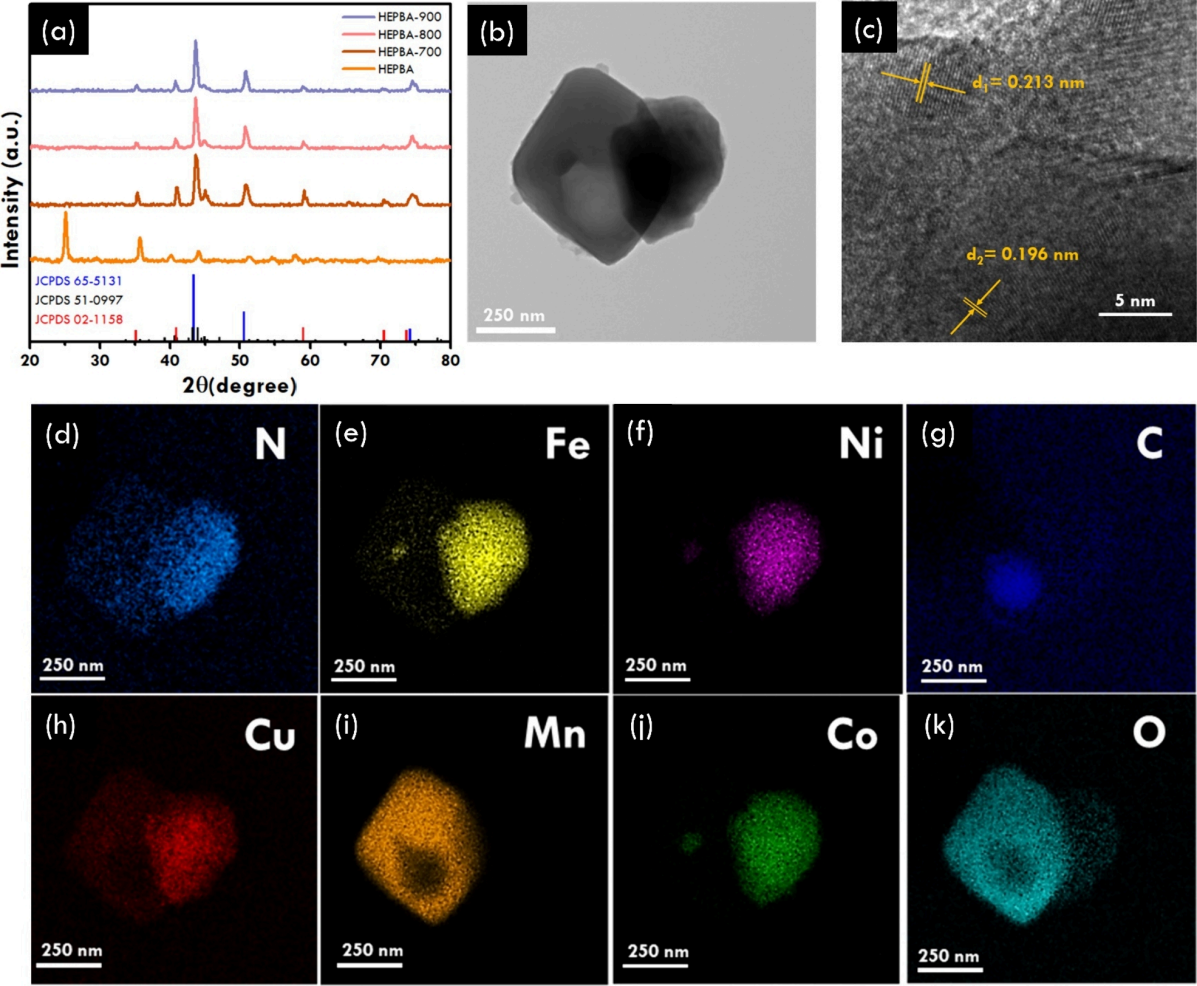
Characterization of HEPBA and its derivation. (a) XRD pattern of annealed HEPBA at various temperatures; (b) High resolution TEM image of HEPBA-800 and (c) the corresponding TEM lattice fringe; EDS elemental mapping images of (d) N, (e) Fe, (f) Ni, (g) C, (h) Cu, (i) Mn, (j) Co, and (k) O for HEPBA-800.

Several characterizations were employed to comprehend the effects of annealing on the morphology and elemental composition of HEPBA and their derived nanoparticles. The XRD technique employed to observe the structural phase of annealed HEPBA is shown in Figure 2a. All of the XRD patterns of annealed HEPBA revealed significant peaks at 43.4°, 50.6°, and 74.3°, which are indexed to the face-centered cubic phase of Fe_3_Ni_2_ (JCPDS card no. 65-5131) with a slight shift due to the existence of the other transition metals combining into the multimetallic alloy state (Fe, Ni, Cu, and Co). The diffraction peaks of annealed HEPBA also show minor peaks at 35.7°, 40.7°, 43.3°, 43.9°, and 47.1°, which are indexed to Fe_5_C_2_ (JCPDS card no. 51-0997), and 35.1°, 40.9°, 59.1°, 70.6°, and 73.8°, which are indexed to MnO (JCPDS card no. 02-1158), suggesting the formation of transition-metal carbide and manganese oxide, respectively.

This result suggests that the HEPBA underwent a complete transformation into exquisite multi (multimetallic alloy, metal carbide, and metal oxide) heterostructure nanoparticles following the annealing process, based on the disappearance of the PBA structure’s diffraction peaks. Transmission electron microscopy (TEM) images of annealed HEPBA-800 are shown in Figure 2b, which displays a HEPBA morphology that exhibits an irregular shape with a particle size of 400 nm. The SEM image in Figure S2 further clarifies the irregular shape of the resulted HEPBA-800. To acquire the microstructure, one of the HEPBA-800 was chosen randomly for high-resolution TEM analysis, and a lattice fringe image was gathered (Figure 2c). The lattice fringes with spacings of *d*_1_ ≈ 0.213 nm and *d*_2_ ≈ 0.196 nm are observed, corresponding to the face-centered-cubic (FCC) structure (111) planes and cubic Co_3_C structure (112) planes, respectively (intensity profile in Figure S3), further confirming the dual existence of multimetallic alloy and carbide in this structure.^27−29^ The EDS elemental mapping images (Figure 2d–k) indicated a uniform element distribution of Fe, Ni, Cu, Co, and Mn. Interestingly, we found high intensities of Mn and O in the same spot, implying Mn oxide formation. The ICP-OES results from Table S1 show a notable increase in relative metal concentrations after pyrolysis. This increase is attributed to the reduction in carbon content during pyrolysis, which concentrates the metals in the final structure. The ICP-OES data indicate a uniform distribution of all elements throughout HEPBA, confirming the successful formation of the material. The actual atomic ratios for each element in the as-synthesized HEPBA were Fe_0.14_Co_0.33_Mn_0.11_Cu_0.11_Ni_0.30_, yielding a mixed configurational entropy (Δ*S*_mix_^conf^) of 1.6*R*, and thus based on the entropy definition, it can be regarded as high-entropy (Δ*S*_mix_^conf^ larger than 1.5*R*).^30^ After pyrolysis, these ratios were adjusted to Fe_0.44_Co_0.13_Mn_0.17_Cu_0.12_Ni_0.13_, with a calculated configurational entropy (Δ*S*_mix_^conf^) of 1.45*R*, indicating that the material no longer retains its high-entropy properties postpyrolysis. Despite these compositional shifts, the uniform distribution of elements was maintained, indicating that the structural integrity of the material remains intact.

### 3.3 Local Coordination Investigation

**Figure 3:**
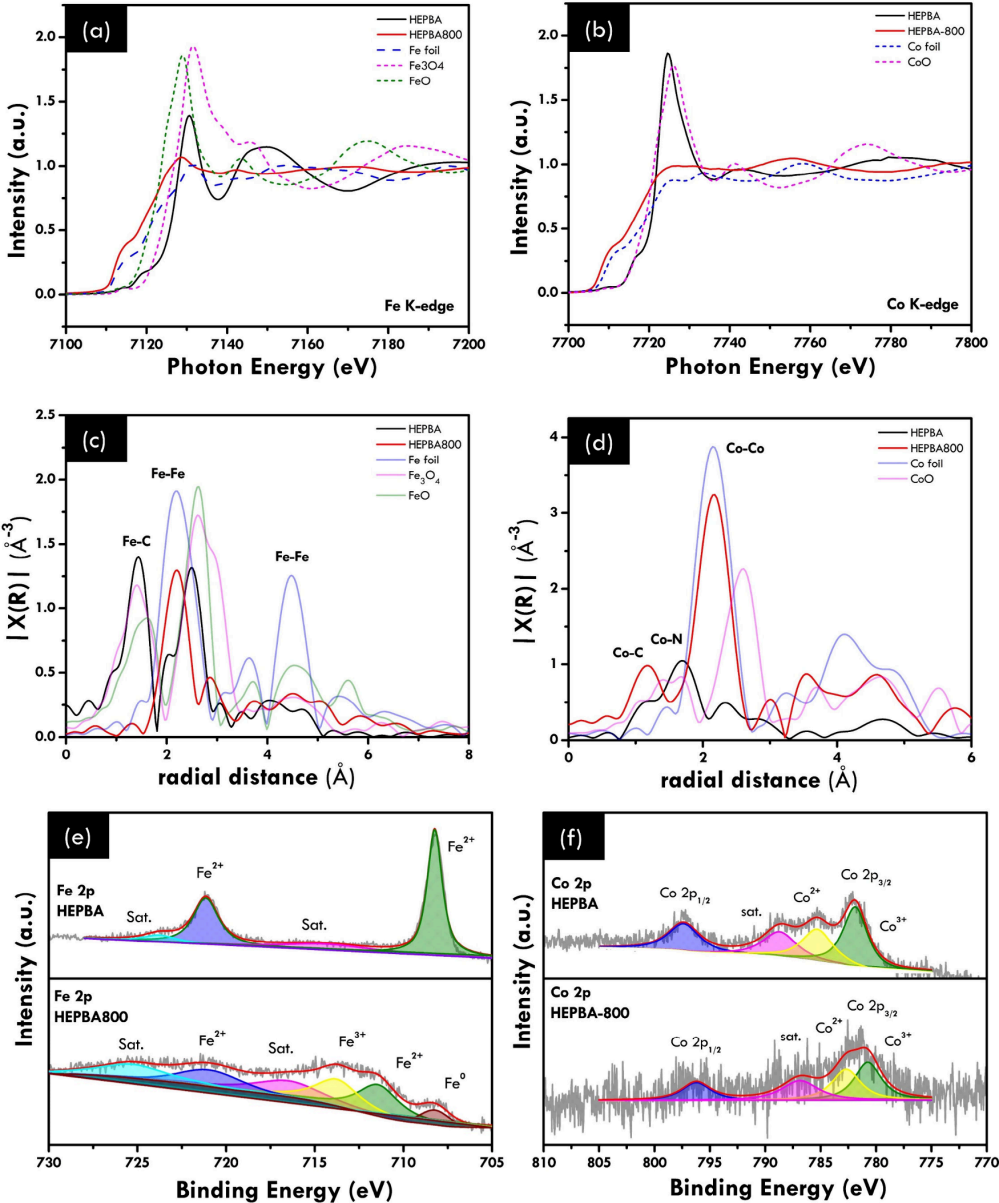
Characterizationof the HEPBA and HEPBA-800 by X-ray absorption (XAS) and X-ray photoelectron spectroscopy (XPS). XAS spectra of HEPBA and HEPBA-800: (a) Fe K-edge and (b) Co K-edge. Corresponding Fourier-transformed EXAFS spectra at (c) Fe K-edge and (d) Co K-edge; High-resolution XPS spectra of HEPBA and HEPBA-800: (e) Fe 2p and (f) Co 2p.

X-ray absorption spectroscopy (XAS) further studied the formation of HEPBA-800 in comparison to HEPBA. The oxidation state and the local coordination environment were presented. The Fe K-edge and Co K-edge XANES (Figures 3a and 3b) demonstrate that after annealing, both Fe and Co in HEPBA-800 underwent a shift from 2+ to 0 valence states, indicating that both Fe and Co primarily exist in a metallic state. The post-edge for Fe and Co in HEPBA-800 demonstrated minor variance in form and intensity compared to the reference metal Fe and Co foils. The above results suggest the formation of an alloy rather than fracture of elements, as evidenced by the absence of equivalent lengths observed in metal Fe and Co foils. The Ni K-edge XANES spectra also reveal results familiar to Co (Figure S4a). The Mn and Cu K-edge XANES, as shown in Figure S4b,c, indicate that Mn and Cu primarily existed in the Mn^2+^ and Cu^2+^ valence states, respectively. Furthermore, the Fourier-transformed extended X-ray absorption fine structure (FT-EXAFS) of HEPBA displays Fe coordinates with six carbon atoms, resulting in an Fe–C bond length of 1.44 Å (Figure 3c). This shift further validates the high-entropy characterization, when compared to the bimetallic PBA in the literature.^31^ Additionally, Figure 3d and Figure S4d–f display a 6-fold coordination structure of Co, Ni, Cu, and Mn, each with the closest Co–N, Ni–N, Cu–N, and Mn–N bonds, respectively. Moreover, the Fe K-edge FT-EXAFS spectrum for HEPBA-800 reveals obvious Fe–Fe (Figure 3c), further suggesting that Fe is successfully transformed to alloy and carbide from the PBA framework, which is confirmed by the change of the PBAs peak (Fe–C) to the alloy peak (Fe–Fe). Meanwhile, the Co K-edge of HEPBA-800 in Figure 3d shows that the first shell of the Co center, which belongs to the Co–C bonds, illustrated an increasing peak at a radial distance (*R*) of 1.17 Å. This may indicate a transformation of the PBA framework (Co–N) into a transition metal carbide (Co–C). Additionally, the outer shell, corresponding to Co–Co bonds at 2.17 Å, suggests a transition from the Co–N bonds in the PBA framework to an alloy. A similar result was observed in the Fourier-transformed EXAFS of Ni and the Cu K-edge spectrum for HEPBA-800 with the different peak ratios of Ni–C, Cu–C and Ni–Ni, Cu–Cu, respectively (Figure S4a,c,d,f). Meanwhile, the EXAFS of Mn shows that the highest peak of HEPBA 800 which belongs to the Mn–O–Mn bond has the exact same position with the reference Mn_2_O_3_.^32^ This finding indicates that the formation of Mn oxide highly likely exists. Thus, the combination of these results still suggest that the combination of multimetallic alloy, metal carbide, and metal oxide was successfully derived from HEPBA.

Chemical surface analysis used XPS to identify the active elemental bonding and detect the oxidation state in HEPBA-800. The XPS full scan spectra (Figure S5a) show that HEPBA and HEPBA-800 contain elements (C, O, N, Fe, Ni, Co, Mn, and Cu). The full scan result indicates that five metal elements involved in catalyst HEPBA-800 were found on its surface, aligning with the mapping results. Furthermore, non-metallic elements (C, N, and O) originating from the PBA framework and functional group were detected. The Fe 2p spectra displayed in Figure 3e exhibit two doublets of Fe 2p_1/2_ and Fe 2p_3/2_, which reveal the Fe^3+^/Fe^2+^ peaks at 711.5/713.8 eV and the Fe^2+^ peak at 721.1 eV, respectively. The Fe^0^ peak (metal state) at 708.3 eV and a satellite peak at 713.7 eV also appear in the Fe 2p spectra. These results are aligned well with the EXAFS result. The elevated valence state of Fe^3+^ has the potential to facilitate the transformation of other metal oxidation states, resulting in accelerated oxygen evolution reaction (OER) kinetics.^16^ The Co 2p spectra (Figure 3f) exhibited binding energies of 796.3 eV in the Co 2p_1/2_ spectrum. The corresponding Co peaks were also displayed at 786.9, 782.7, and 780.8 eV in the satellite Co, Co^2+^, and Co^3+^ peaks, respectively, in the Co 2p_3/2_ spectrum. The detailed analysis of the high-resolution XPS of Ni 2p, Mn 2p, and Cu 2p spectra is illustrated in Figure S5b–d. The Ni 2p spectra indicate that Ni in HEPBA-800 exhibits two states: Ni^2+^ (873.0 and 855.6) and Ni^3+^(857.3 and 875.3 eV) (Figure S5b). The Mn 2p spectra indicate that Mn in HEPBA-800 exhibits two states: Mn^3+^ (641.2 and 649.3 eV) and Mn^4+^(643.5 and 655.3 eV) (Figure S5c). The Cu 2p spectra indicate that Cu in HEPBA-800 exhibits two states: Cu^0^ (933.3 and 953.2 eV) and Cu^2+^(934.8 and 954.9 eV) (Figure S5d). As a result of XPS, catalyst HEPBA-800 exhibits numerous oxidation states for each metal element. The metallic state of Fe and Cu results from their transformation into multimetallic alloy and likely occurs because of the high surface energy, leading to partial surface oxidation.^33,34^ This transformation could induce numerous oxidation states in each element, thus contributing to the complex electronic properties of the high-entropy-derived material. Moreover, the absence of the metallic state of Mn, which is related to the XAS data, further confirms the carbide covering of all alloy parts and the presence of Mn oxide. Therefore, based on these identifications, we can fairly confirm the structure and composition of exquisite multiphase (multimetallic alloy, metal carbide, and metal oxide) heterostructure nanoparticles.

### 3.4 Electrochemical Performance of HEPBA and Their Derived Samples at Different Temperatures

**Figure 4:**
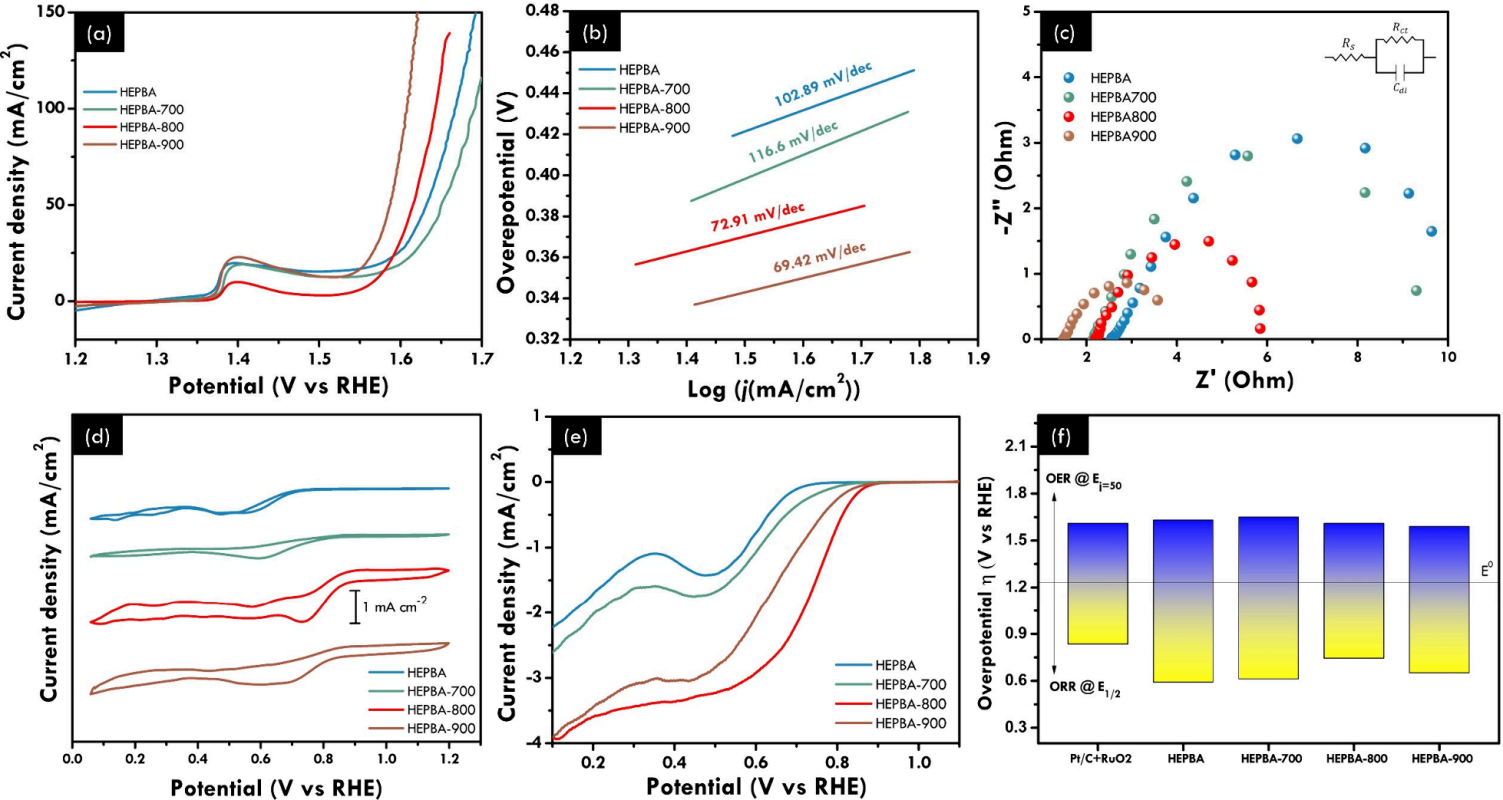
Electrochemical performance of HEPBA and derived samples at different temperatures. (a) OER polarization curves of HEPBA, HEPBA-700, HEPBA-800, and HEPBA-900 in the O_2_ saturated 1 M KOH solution. (b) Tafel slope from the OER LSV curves. (c) Electrochemical impedance spectra (EIS). (d) Cyclic voltammetry of HEPBA, HEPBA-700, HEPBA-800, and HEPBA-900 samples using the RDE setup in O_2_-saturated 0.1 M KOH. (e) ORR polarization curves of HEPBA, HEPBA-700, HEPBA-800, and HEPBA-900 under 1600 rpm. (f) Comparison of corresponding potential gap (*E*_1/2_ and *E*_η50_) from the polarization curves.

The investigation into the electrocatalytic activities for ORR and OER of HEPBA and annealed HEPBA, subjected to temperatures ranging from 700 to 900 °C while maintaining a constant atomic ratio, was conducted in the three-electrode electrochemical system. The investigation of the oxygen evolution reaction (OER) performance utilized Ni foam as a substrate for the working electrode, resulting in the appearance of a redox peak attributed to Ni. However, this presence did not affect the performance, as demonstrated by the performance of the OER of Ni foam illustrated in Figure S6. Figure 4a displays the OER polarization curves, indicating that HEPBA treated at 800 °C (HEPBA-800) demonstrates a low overpotential of 340 mV at 10 mA cm^–2^, which is slightly higher than that of HEPBA-900 (η_10_ = 310 mV). HEPBA-900’s outstanding OER is due to the higher content of the alloys, indicating that the multimetallic alloys enhance the OER performances. Corresponding to the OER polarization curves, the Tafel slope in Figure 4b was utilized to demonstrate the kinetic activity, revealing that HEPBA-800 exhibited a notably low slope of 72.91 mV dec^–1^. The obtained outcome exhibits a notable current density with low overpotential, indicating that the annealing modification effectively facilitated the mass transfer process of the activated metal.^35^ The charge-transfer resistance (*R*_ct_) further clarifies the superior intrinsic activity of HEPBA-900 compared to other samples by exhibiting a significantly smaller semicircular diameter in the EIS than HEPBA-800, HEPBA-700, and HEPBA. It suggests that the annealing temperature can significantly improve electrocatalyst performance by controlling the transformation between transition-metal carbide, alloy, and oxide (Figure 4c). The electrocatalytic performance of HEPBA-derived samples toward ORR was evaluated in the O_2_-saturated 1 M KOH solution using the RDE setup. Figure 4d shows that cyclic voltammetry possessed superior ORR activity by HEPBA-800, as evidenced by the greater current density and positive oxygen reduction peak potential. This finding aligns with the ORR polarization curves observed at 1600 rpm (Figure 4e); HEPBA-800 shows a half-wave potential (*E*_1/2_) of 0.74 V and an onset potential of 0.84 V (vs RHE). As confirmed by our XRD, TEM, and XAS results and previous studies, manganese oxides improve transfer charges and metal carbide has excellent electrical and electronic properties.^36,37^ These factors help HEPBA-800 achieve its superior ORR performance, making more active sites available compared with HEPBA-700 and HEPBA-900. The corresponding potential gap comparison between HEPBA, HEPBA-700, HEPBA-800, HEPBA-900, and Pt/C+RuO_2_ is illustrated in Figure 4f. The OER and ORR have remarkable catalytic performance, exhibiting a strongly distorted lattice and modulated surface electronic states due to the high-entropy property.^38^ On the other hand, HEPBA-700’s poor performance on both OER and ORR was attributed to the higher content of Mn oxide, which has poor electrical conductivity, as confirmed by XRD and higher *R*_ct_ value by EIS results. However, the appropriate content of Mn oxide leads to higher OER and ORR performance, as shown by HEPBA-800. Additionally, the samples of HEPBA and HEPBA-800 are selected to compare the activation energy required toward the individual OER and ORR. We used the EIS data at the same overpotential and varied temperatures (25, 30, 40, 50, and 60 °C).^19^ The applied potentials for the OER and ORR are 1.51 and 0.73 V vs RHE. As we can see from the Arrhenius plot in Figures S7a and S7b, the HEPBA needs more activation energy for the OER and ORR compared to HEPBA-800. This result could support that the more active sites are available for HEPBA-800 to accelerate the OER and ORR. Thus, the energy needed to overcome by the HEPBA-800 is less than the pristine HEPBA.

### 3.5 Electrochemical Performance of HEPBA/CNT-*T*

**Figure 5:**
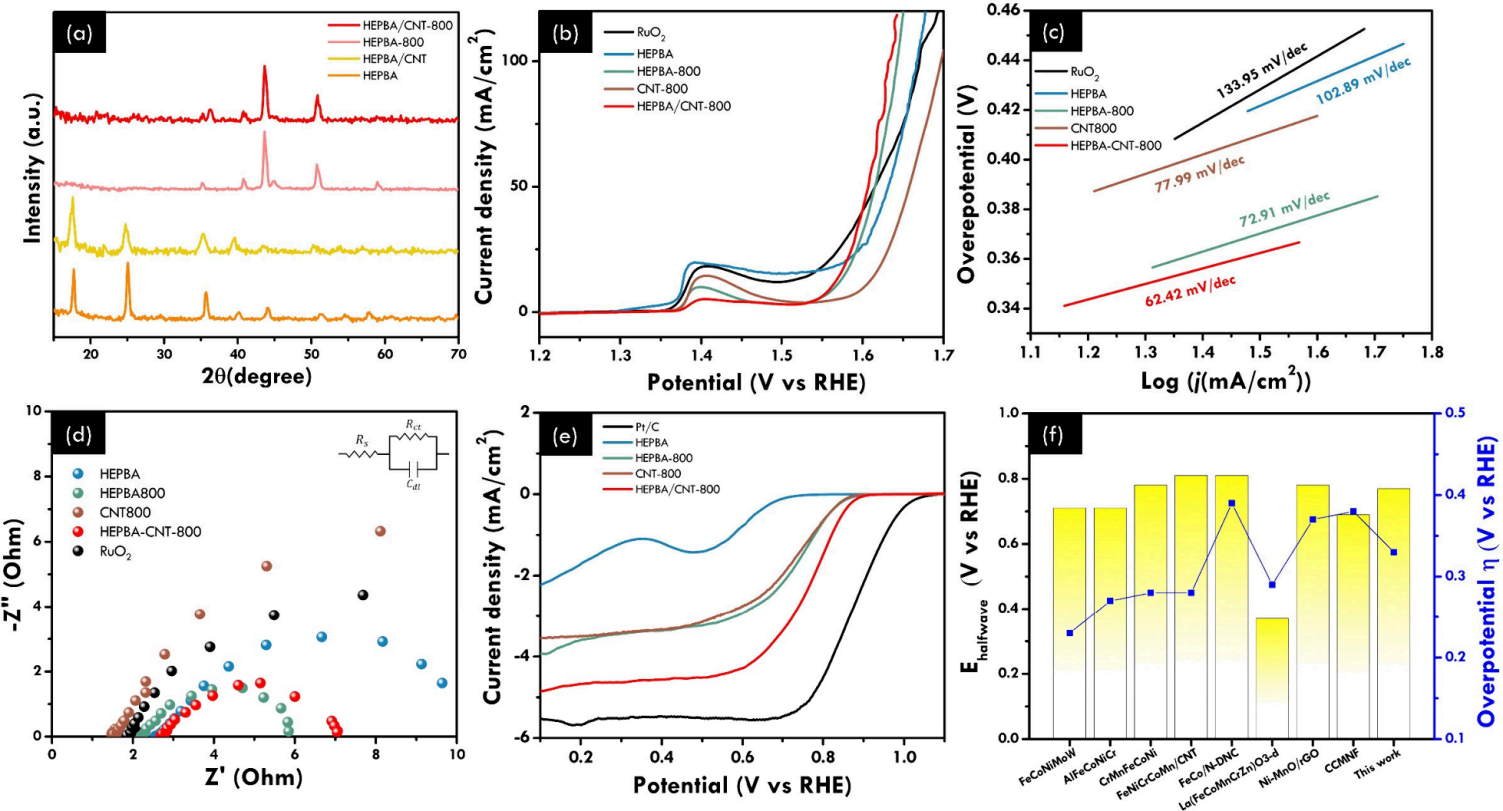
Electrochemical performance of HEPBA/CNT and HEPBA/CNT-*T*. (a) XRD patterns of HEPBA, HEPBA/CNT, HEPBA-800, and HEPBA/CNT-800. (b) OER polarization curves of HEPBA, HEPBA-800, CNT-800, HEPBA/CNT-800, and RuO_2_ in the O_2_-saturated 1 M KOH electrolyte. (c) Tafel slope from the OER LSV curves. (d) Electrochemical impedance spectra (EIS). (e) ORR polarization curves of HEPBA, HEPBA-800, CNT-800, NCNT, HEPBA/CNT-800, and Pt/C using the RDE system in O_2_-saturated 0.1 M KOH under 1600 rpm rotation. (f) Comparison of the bifunctional electrocatalysts based on HE-material reported in partial literatures.

Carbon nanotubes (CNTs) are added to the coprecipitation process to further improve the bifunctional performance, especially ORR, by enhancing electron transmission and reducing the interfacial resistance at the contact area between the current collector and cathode material.^25,39,40^ The process of heterogeneous nucleation and HEPBA growth on the functionalized carbon nanotubes (f-CNTs) interconnects HEPBA, and it is possible that the presence of CNTs impedes composite aggregation, thereby enhancing electrolyte contact in HEPBA while HEPBA is still present. According to the growth mechanism, the bonding can be conceptualized as an intermolecular force (van der Waals).^16,27^ Addition characterization was employed to observe the structural phase of f-CNTs, as shown in Figure S8. Subsequently, we follow the previous annealing procedure to obtain the integration of CNT into HEPBA-derived nanoparticles at *T* °C, which is denoted as HEPBA/CNT-*T*. In Figure 5a, the XRD patterns indicate that all observed diffraction peaks of HEPBA/CNT can be assigned to the typical HEPBA. The absence of characteristic peaks associated with CNTs occurs due to the relatively low concentration of CNTs and the presence of highly crystalline PBA, aligning with existing literature. Nevertheless, the SEM image (Figure S9) displays an interconnected between HEPBA and CNT in the HEPBA/CNT derived nanoparticles at 800 °C (HEPBA/CNT-800), further confirming the successful incorporation of f-CNTs into HEPBA frameworks, and investigates the impact of pyrolysis treatment on the morphology.

The OER data presented in Figure 5b show that the HEPBA/CNT-800 achieves a current density of 10 mA cm^–2^ at an overpotential of 330 mV, outperforming HEPBA-800, which requires an overpotential of 340 mV. This improvement is further explored through the kinetic activity represented by the Tafel slope in Figure 5c, showing that HEPBA/CNT-800 exhibited the slope value of 62.42 mV dec^–1^. The small *R*_ct_ highlights the enhanced intrinsic activity of HEPBA-800 and HEPBA/CNT-800, which demonstrates that the transformation of HEPBA significantly boosts charge transfer kinetic activity, as shown in Figure 5d. The Nyquist plot of the HEPBA/CNT-800 also shows a nearby semicircular diameter. This result implies that CNT does not primarily enhance OER performance by facilitating electron transfer and ion transport at the interface. On the other hand, the remarkable ORR performance shows an onset potential of HEPBA/CNT-800 is 0.87 V (vs RHE) with the half-wave potential (*E*_1/2_) of 0.77 V (vs RHE) in Figure 5e, which is attributed to the improvement in catalytic performance resulting from introduced CNT. Additionally, as shown in Figure 5f, HEPBA/CNT-800 was compared with other HE concepts and related electrocatalysts, and detailed information is shown in Table S2. Next, to optimize the HEPBA/CNT-800 performance, the bifunctional electrocatalyst of HEPBA/CNT-800 with different weight ratios is shown in Figure S10. The results suggest that as the CNT concentration increased, the interconnected CNTs act as an effective composite for enhanced conductivity, distribution of active sites, and improved ORR activity.

### 3.6 Electrochemical Performance of Rechargeable Aqueous Zn–Air Batteries

**Figure 6:**
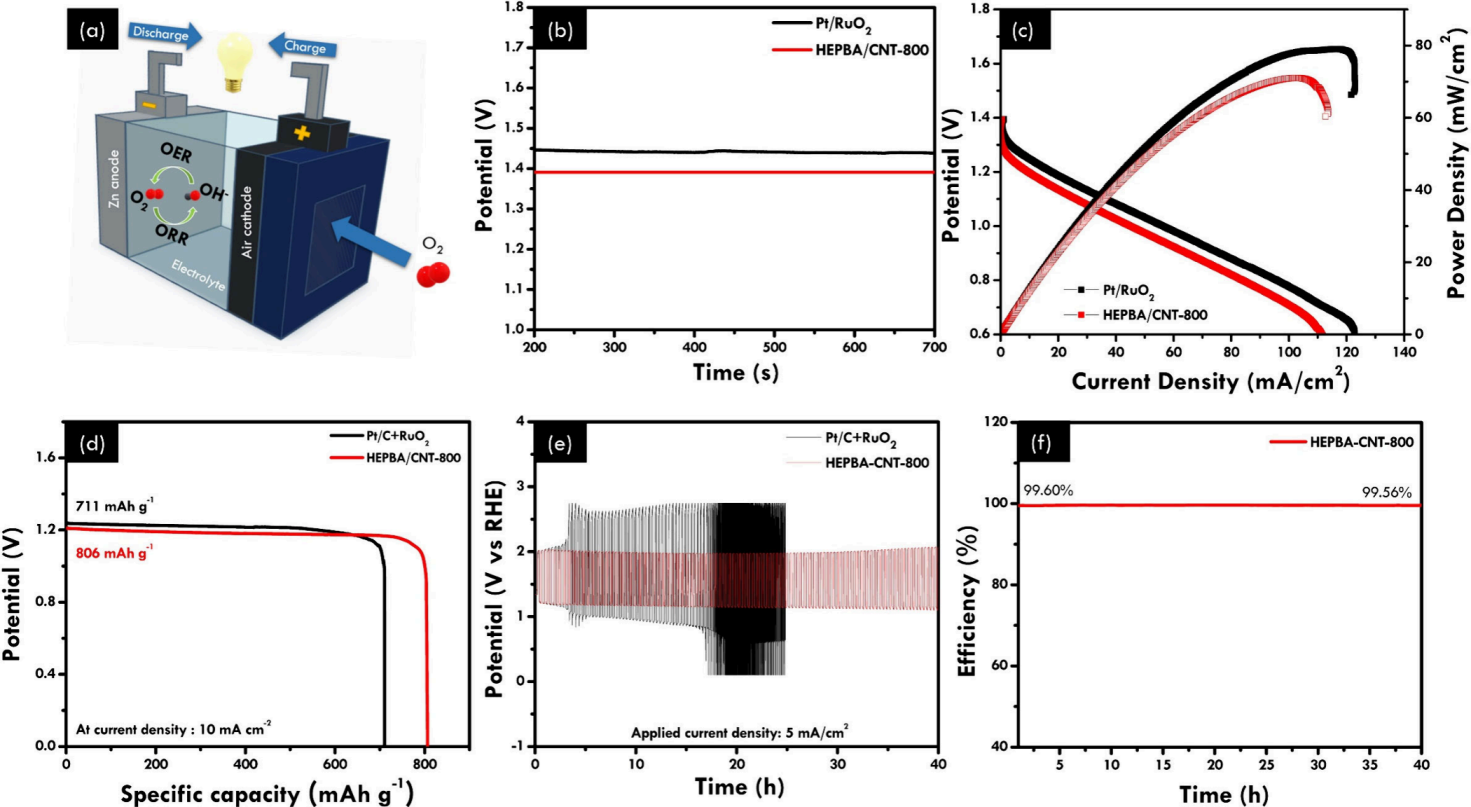
Aqueous rechargeable zinc–air battery performance of the HEPBA/CNT-800. (a) Schematic structure of liquid rechargeable ZABs. (b) Open circuit potential between HEPBA/CNT-800 and Pt/C+RuO_2_. (c) Power density and discharge polarization curves between HEPBA/CNT-800 and Pt/RuO_2_. (d) Specific capacities of ZABs made by the HEPBA/CNT-800 and Pt/C+RuO_2_ catalysts at 10 mA cm^–2^. (e) Long-term stability test for HEPBA/CNT-800 and Pt/C+RuO_2_. (f) Coulombic efficiency of HEPBA/CNT-800.

To investigate the possibility of HEPBA/CNT-800 catalysts for application in rechargeable ZABs, HEPBA/CNT-800-based rechargeable aqueous ZABs were assembled, as shown in Figure 6a. Rechargeable ZAB-related characterization was carried out and compared with that of Pt/C+RuO_2_. The as-synthesized catalyst solutions were loaded onto carbon paper as air cathodes, while a zinc plate served as the counter electrode. This study used an aqueous solution of 6.0 M KOH, and 0.2 M zinc acetate served as the electrolyte. Figure 6b shows that the HEPBA/CNT-800-based ZAB presents a slightly lower open-circuit voltage (OCV = 1.39 V) than the Pt/C+RuO_2_-based ZAB (1.44 V). Subsequently, ZAB with the HEPBA/CNT-800 air cathode offers a power density of 71 mW cm^–2^ at a current density of 102 mA cm^–2^, which is slightly lower than ZAB using Pt/C+RuO_2_ (75 mW cm^–2^ at 105 mA cm^–2^) (Figure 6c). Furthermore, the specific capacity of HEPBA/CNT-800-based ZAB is up to 806 mAh g^–1^ at 10 mA cm^–2^ (Figure 6d), which is significantly higher compared to devices assembled with Pt/C+RuO_2_ as catalysts (711 mAh g^–1^), demonstrating exceptional energy storage capability. To determine the stability of the air electrode, a series of discharging and charging cycles at a constant current were carried out, as shown in Figure 6e. HEPBA/CNT-800 had a slight change in voltage after more than 40 h of cycling at a charging/discharging current density of 5 mA cm^–2^. In contrast, Pt/C+RuO_2_ had a significant change in the voltage after less than 20 h of cycling, highlighting the outstanding stability of HEPBA/CNT-800. Furthermore, HEPBA/CNT-800 also demonstrated a high Coulombic efficiency, as shown in Figure 6f. The comparison of HEPBA/CNT-800 and the bifunctional electrocatalysts based on the HE-concept and related materials is reported in the partial literature, as shown in Table S3. This work’s catalysts exhibit good performance and are comparable to others in the fields of electrocatalytic performance and RZAB performance. These cell parameters suggest that the catalyst HEPBA/CNT-800 has excellent potential for application with rechargeable ZABs and might also be considered for sustainable energy storage technologies.

## 4 Conclusion

In summary, we have successfully fabricated HEPBA-derived heterostructure nanoparticles as promising electrode materials for the bifunctional oxygen catalyst. The HEPBA acts as the efficient template for the bifunctional reaction, resulting in the multimetallic transition-metal alloy, while the C and N elements from the cyanide group combine with the metal in the PBA framework to form transition-metal carbide. The XRD patterns, elemental mapping, hard XAS, and XPS data confirm the successful formation of 3-phase heterostructures, consisting of transition-metal alloys, carbides, and oxides. According to the suitable temperature, controlling the formation of the alloy and carbide in the HEPBA framework leads to improved OER and ORR activity. The elusive multiphase heterostructure nanoparticles manifested two active sites for selective ORR and OER according to their synergistic effect as well as conductivity and mass transfer. HEPBA/CNT-800 exhibits an OER overpotential of 330 mV at 10 mA cm^–2^ in 1 M KOH, an ORR half-wave potential of 770 mV in 0.1 M KOH, and a bifunctional oxygen overpotential Δ*E* of only 0.79 V, which is superior to other HEPBA-derived samples under the same conditions. The aqueous RZAB with HEPBA/CNT-800 as the cathode showed an open-circuit voltage of 1.39 V and provided a high energy density of 71 mW cm^–2^ under a current density of 102 mA cm^2^ and can be charged and discharged in cycles up to 40 h, revealing good stability under an applied current density at 5 mA cm^–2^ and also exhibits a discharge specific capacity of 806 mAh g^–1^ at a current density of 10 mA cm^–2^, demonstrating its excellent potential for application with rechargeable ZABs and might also be considered for sustainable energy storage technologies.

## Supporting Information Available

Comprehensive figures elucidating the intricate characterization and electrochemical performance of the materials obtained in this study (PDF)
