# Supplementary material for: High-Entropy Prussian Blue Analogue Derived Heterostructure Nanoparticles as Bifunctional Oxygen Conversion Electrocatalysts for the Rechargeable Zinc–Air Battery
Source: ACS Appl Mater Interfaces. 2024 Nov 4;16(45):62022–32. doi: 10.1021/acsami.4c13387 (PMC11565479; doi:10.1021/acsami.4c13387)
Supplement: Supplementary file 1 — am4c13387_si_001.pdf [file am4c13387_si_001.pdf]

Supporting Information

High-entropy Prussian Blue Analogue derived Heterostructure Nanoparticles as Bifunctional Oxygen Conversion Electrocatalyst for Rechargeable Zinc-air Battery

Wuttichai Tanmathusorachai[1#], Sofiannisa Aulia[1#], Mia Rinawati[1], Ling-Yu Chang[2], Chia-Yu Chang[3], Wei-Hsiang Huang[4], Ming-Hsien Lin[5,6], Wei-Nien Su[3,6], Brian Yuliarto[7], and Min-Hsin Yeh[1,6*]

[1] *Department of Chemical Engineering, National Taiwan University of Science and Technology, Taipei 10607, Taiwan*

[2] *Department of Chemical Engineering and Biotechnology, National Taipei University of Technology, Taipei 10608, Taiwan*

[3] *Graduate Institute of Applied Science and Technology, National Taiwan University of Science and Technology, Taipei, 10607, Taiwan*

[4] *National Synchrotron Radiation Research Center, Hsinchu, 30076, Taiwan*

[5] *Department of Chemical and Materials Engineering, Chung Cheng Institute of Technology, National Defense University, Dasi, Taoyuan 335, Taiwan*

[6] *Sustainable Electrochemical Energy Development Center, National Taiwan University of Science and Technology, Taipei 10607, Taiwan*

[7] *Advanced Functional Materials Laboratory, Department of Engineering Physics, Institute of Technology Bandung (ITB), Bandung, 40132, Indonesia*

[#] *W. Tanmathusorachai and S. Aulia contributed equally to this work.*

[*]Corresponding author: Tel.: +886-2-2737-6643; E-mail: mhyeh@mail.ntust.edu.tw

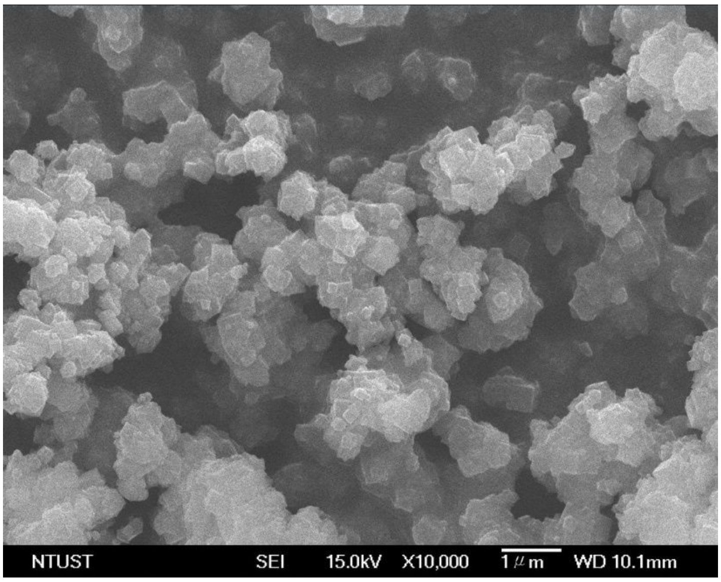

Figure S1 SEM image of lower magnification of as-synthesized HEPBA.

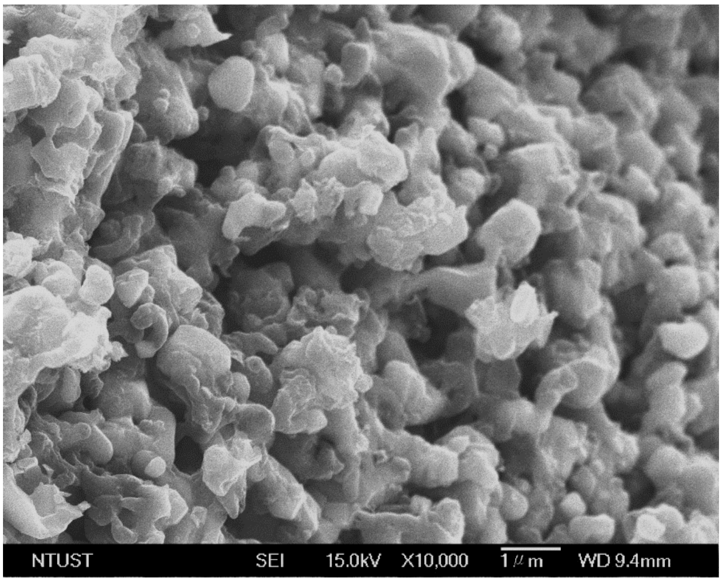

Figure S2 SEM image of lower magnification of as-synthesized HEPBA-800.

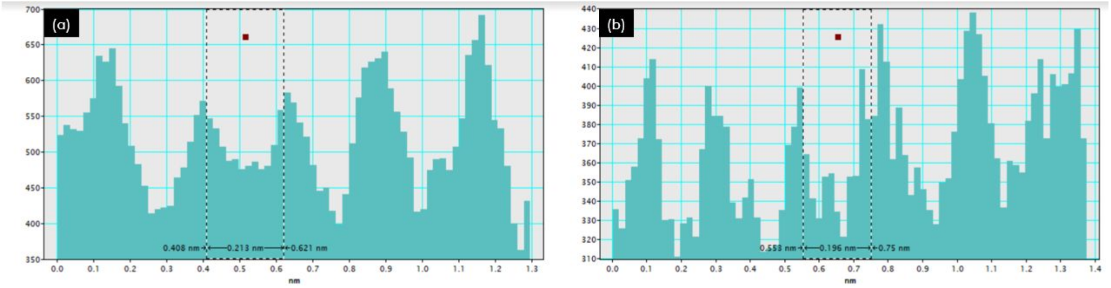

Figure S3 Intensity line profile of Figure 2b. lattice distance of (a) d_1 = 0.213 nm and (b) d_2 = 0.196 nm, corresponding to the (111) lattice fringes of high-entropy alloy and (112) lattice fringes of Co_3C, respectively.

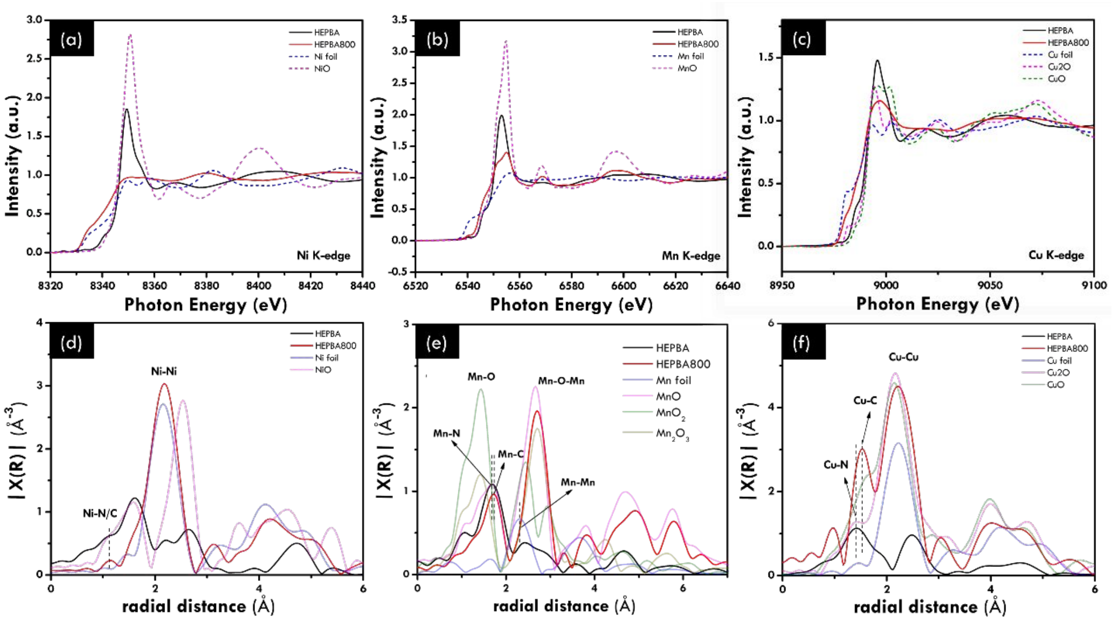

Figure S4 X-Ray absorption (XAS) of the HEPBA and HEPBA-800 (a) Ni K-edge (Ni 2p), (b) Mn K-edge (Mn 2p) spectra, and (c) Cu K-edge (Cu 2p) spectra, Corresponding Fourier-transformed EXAFS at spectra of (d) Ni K-edge, (e) Mn K-edge, and (f) Cu K-edge.

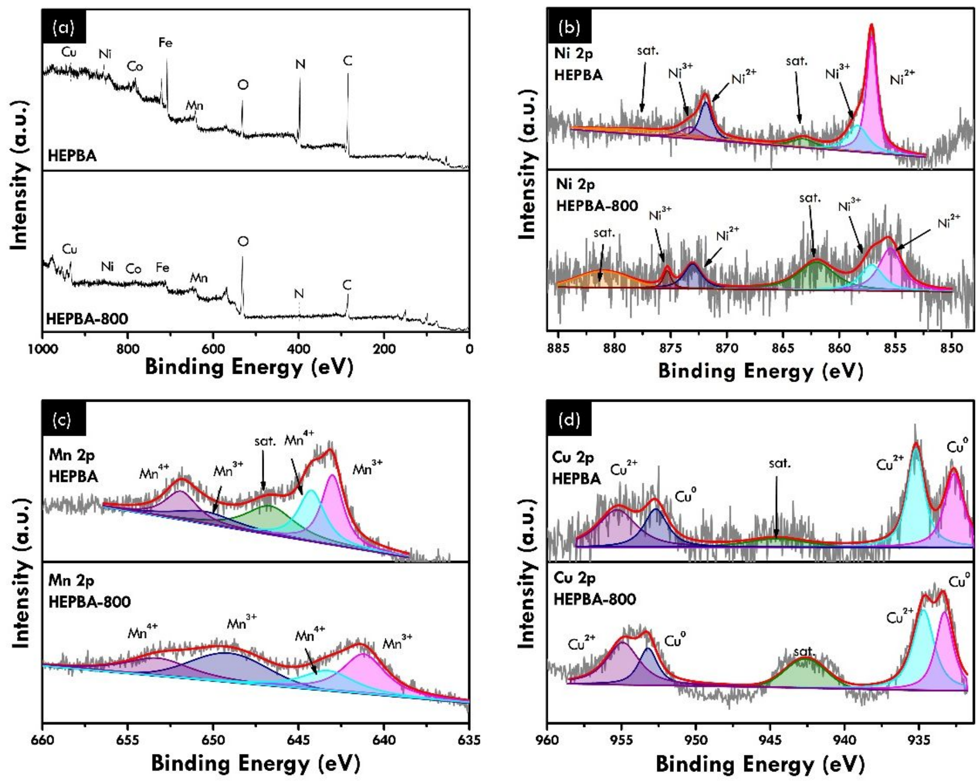

Figure S5 (a) XPS full scan spectra of HEPBA and HEPBA-800, High resolution spectra of (b) Ni 2p, (c) Mn 2p, and (d) Cu 2p.

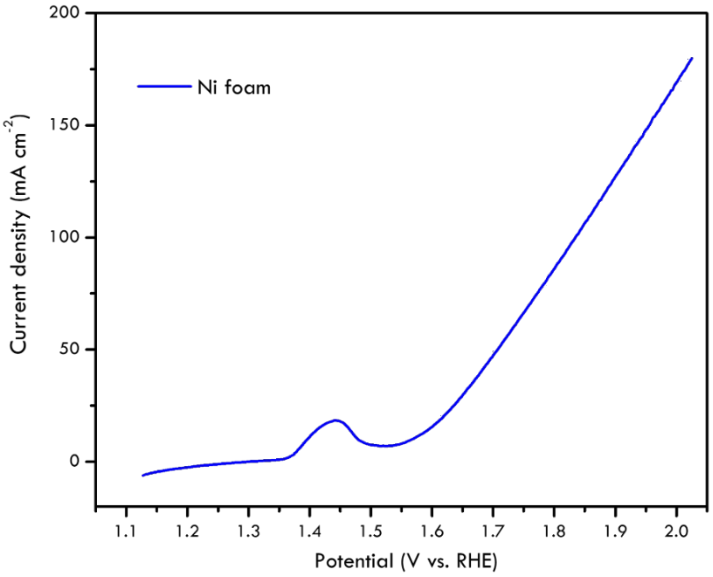

Figure S6 OER polarization curve of Ni-foam (substrate) in the O_2-saturated 1 M KOH electrolyte.

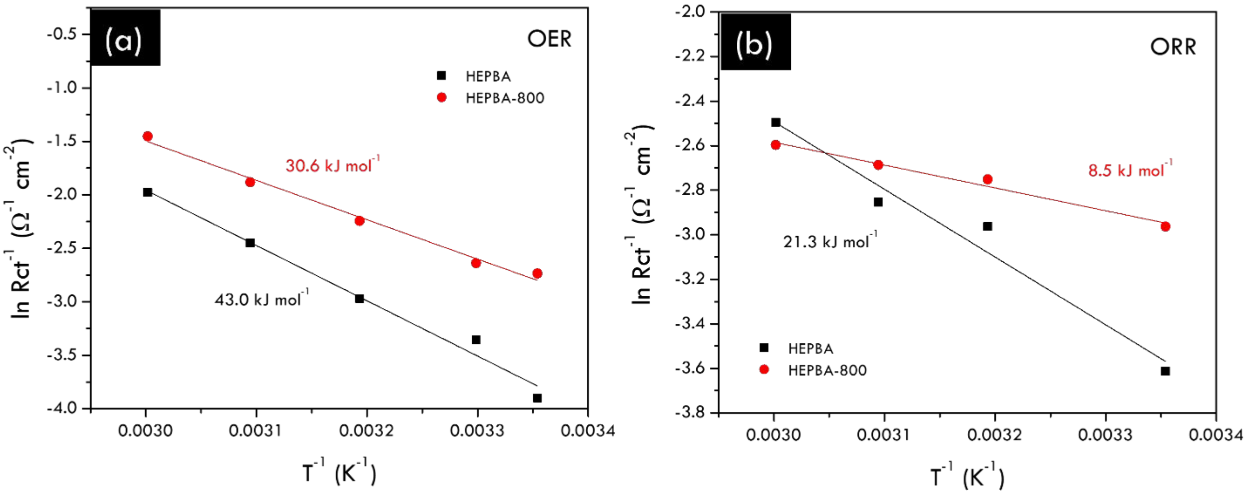

Figure S7 (a) and (b) Arrhenius plot of the OER and ORR from HEPBA and HEPBA-800 samples.

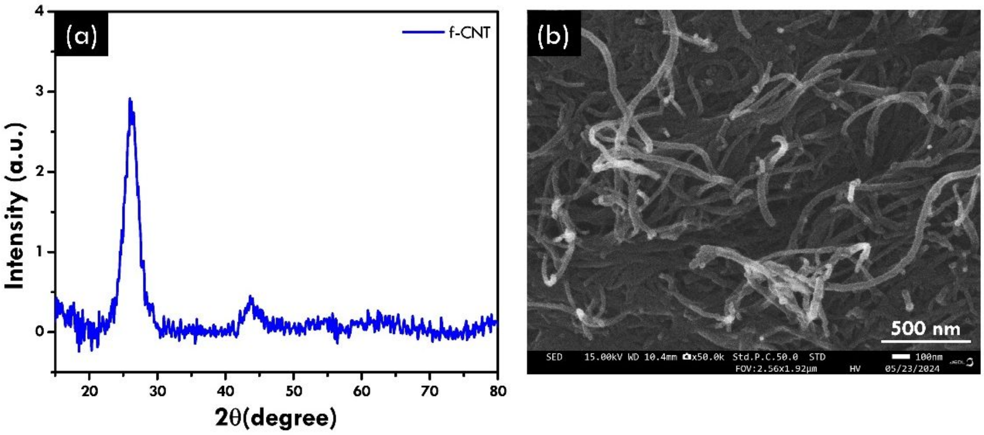

Figure S8 Characterization of functionalized carbon nanotubes (f-CNTs) (a) XRD pattern, (b) SEM image.

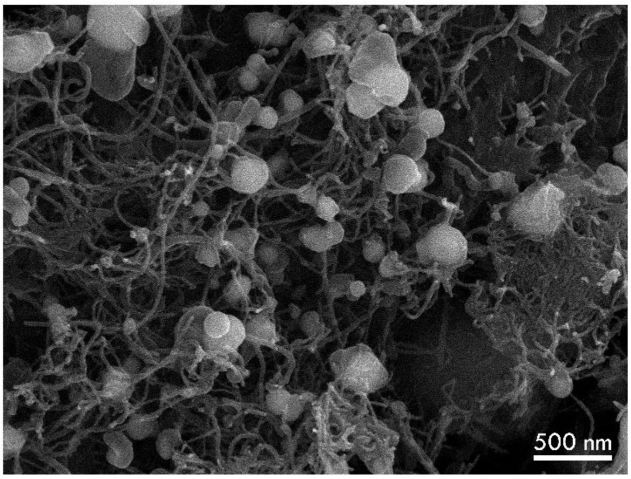

Figure S9 SEM image of the HEPBA/CNT-800.

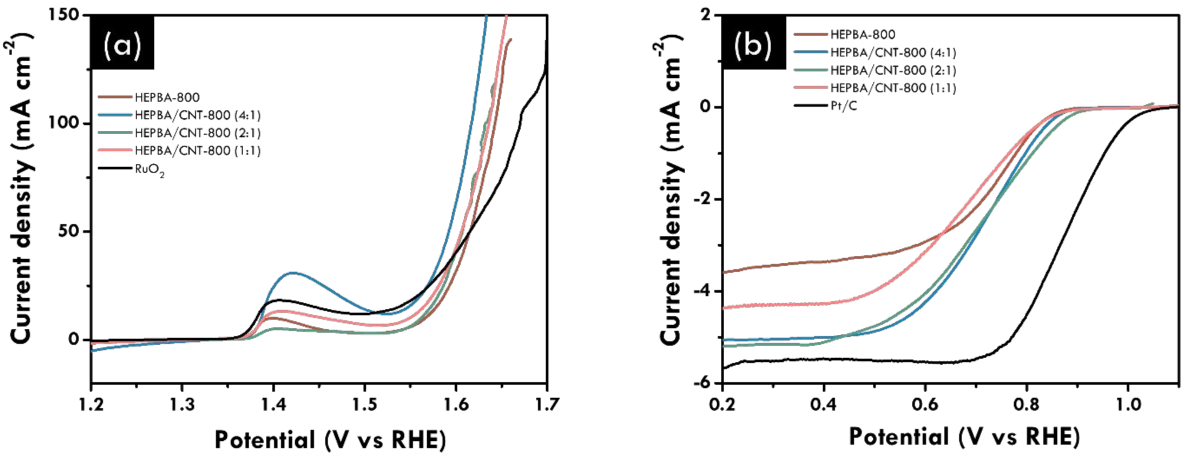

Figure S10 (a) OER polarization curve of HEPBA-800, HEPBA/CNT-800 4:1, 2:1, 1:1, and RuO_2 in the O_2-saturated 1 M KOH electrolyte, (b) ORR polarization curve of HEPBA-800, HEPBA/CNT-800 4:1, 2:1, 1:1, and Pt/C in the O_2-saturated 0.1 M KOH electrolyte at 1600 rpm.

Table S1 Metallic elemental composition of HEPBA and HEPBA-800.

Electrocatalyst	Atomic ratio of metallic elements (at.%)[a]				
	Fe	Co	Mn	Cu	Ni
HEPBA	14.24	33.35	11.07	11.04	30.29
HEPBA-800	44.62	13.19	17.14	11.68	13.36

[a] data obtained by ICP-OES.

Table S2 Bifunctional activity comparison of HE-based bifunctional catalyst reported in the literatures.

**Electrocatalyst**	$\boldsymbol{\eta_{10}}$ **(V)**	$\mathbf{E_{1/2}}$ **(V)**	**ΔE (V)**	**Reference**
**AlNiCoRuMo**	1.475	0.875	0.6	1
**AlFeCoNiCr**	1.5	0.71	0.79	2
**CrMnFeCoNi**	1.51	0.78	0.73	3
**FeNiCrCoMn/CNTs**	1.51	0.81	0.7	4
**AlCuNiPtMn**	-	0.94	-	5
**FeCoNiMoW**	1.46	0.71	0.75	6
**FeCo/N-DNC**	1.62	0.81	0.81	7
**CCMNF**	1.61	0.69	0.92	8
$\mathbf{La(Fe_{0.2}Co_{0.3}Mn_{0.1}Cr_{0.2}Zn_{0.2})O_{3-\delta}}$	1.52	0.37	1.15	9
**NiCo@N-C-2**	1.78	0.81	0.97	10
**HEPBA-800**	**1.57**	**0.73**	**0.84**	**This work**
**HEPBA/CNT-800**	**1.56**	**0.77**	**0.79**	**This work**

Table S3 ZAB performances comparison of HE-based bifunctional catalyst reported in the literatures.

Electrocatalyst	Open circuit potential (V)	Power density (mW cm^{-2})	Specific capacity (mAh/gZn)@J (mA cm^{-2})	Stability (h)	Ref.
**AlNiCoRuMo**	1.48	146.5	-	2 mA cm^{-2}, for 120 h	1
**AlFeCoNiCr**	-	125	800@20	2 mA cm^{-2}, for 60 h	2
**CrMnFeCoNi**	1.49	116.5	836@8	12 mA cm^{-2}, for 240 h	3
**FeNiCrCoMn/CNTs**	1.37	128.6	760@10	5 mA cm^{-2}, for 256 h	4
**AlCuNiPtMn**	-	-	831@20	-	5
**FeCoNiMoW**	1.59	116.9	857@8	8 mA cm^{-2}, for 660 h	6
**FeCo/N-DNC**	-	115	804@5	10 mA cm^{-2}, for 34 h	7
**CCMNF**	1.52	16.5	688.6@5	5 mA cm^{-2}, for 33 h	8
**La(Fe0.2Co0.3Mn 0.1Cr0.2Zn0.2)O3−δ**	-	82	390.7@5	-	9
**MCN**	1.53	103.3	801.5@10	10 mA cm^{-2}, for 10 h	11
**FeCo@MNC**	1.41	115	-	20 mA cm^{-2}, for 24 h	12
**HEPBA/CNT-800**	**1.39**	**71**	**806@10**	**5 mA cm^{-2}, for 40 h**	**This work**

References

(1) Qiu, H.-J.; Fang, G.; Gao, J.; Wen, Y.; Lv, J.; Li, H.; Xie, G.; Liu, X.; Sun, S. Noble Metal-Free Nanoporous High-Entropy Alloys as Highly Efficient Electrocatalysts for Oxygen Evolution Reaction. *ACS Materials Letters* **2019**, *1*, 526-533.

(2) Fang, G.; Gao, J.; Lv, J.; Jia, H.; Li, H.; Liu, W.; Xie, G.; Chen, Z.; Huang, Y.; Yuan, Q.; et al. Multi-Component Nanoporous Alloy/(Oxy)Hydroxide for Bifunctional Oxygen Electrocatalysis and Rechargeable Zn-Air Batteries. *Applied Catalysis B: Environmental* **2020**, *268*, 118431.

(3) He, R.; Yang, L.; Zhang, Y.; Wang, X.; Lee, S.; Zhang, T.; Li, L.; Liang, Z.; Chen, J.; Li, J.; et al. A Crmnfeconi High Entropy Alloy Boosting Oxygen Evolution/Reduction Reactions and Zinc-Air Battery Performance. *Energy Storage Materials* **2023**, *58*, 287-298.

(4) Cao, X.; Gao, Y.; Wang, Z.; Zeng, H.; Song, Y.; Tang, S.; Luo, L.; Gong, S. Fenicrcomn High-Entropy Alloy Nanoparticles Loaded on Carbon Nanotubes as Bifunctional Oxygen Catalysts for Rechargeable Zinc-Air Batteries. *ACS Applied Materials & Interfaces* **2023**, *15*, 32365-32375.

(5) Li, S.; Tang, X.; Jia, H.; Li, H.; Xie, G.; Liu, X.; Lin, X.; Qiu, H.-J. Nanoporous High-Entropy Alloys with Low Pt Loadings for High-Performance Electrochemical Oxygen Reduction. *Journal of Catalysis* **2020**, *383*, 164-171.

(6) He, R.; Yang, L.; Zhang, Y.; Jiang, D.; Lee, S.; Horta, S.; Liang, Z.; Lu, X.; Ostovari Moghaddam, A.; Li, J.; et al. A 3d-4d-5d High Entropy Alloy as a Bifunctional Oxygen Catalyst for Robust Aqueous Zinc–Air Batteries. *Advanced Materials* **2023**, *35*, 2303719.

(7) Fu, G.; Liu, Y.; Chen, Y.; Tang, Y.; Goodenough, J. B.; Lee, J.-M. Robust N-Doped Carbon Aerogels Strongly Coupled with Iron–Cobalt Particles as Efficient Bifunctional Catalysts for Rechargeable Zn–Air Batteries. *Nanoscale* **2018**, *10*, 19937-19944.

(8) Madan, C.; Jha, S. R.; Katiyar, N. K.; Singh, A.; Mitra, R.; Tiwary, C. S.; Biswas, K.; Halder, A. Understanding the Evolution of Catalytically Active Multi-Metal Sites in a Bifunctional High-Entropy Alloy Electrocatalyst for Zinc–Air Battery Application. *Energy Advances* **2023**, *2*, 2055-2068.

(9) Erdil, T.; Toparli, C. B-Site Effect on High-Entropy Perovskite Oxide as a Bifunctional Electrocatalyst for Rechargeable Zinc–Air Batteries. *ACS Applied Energy Materials* **2023**, *6*, 11255-11267.

(10) Fu, Y.; Yu, H.-Y.; Jiang, C.; Zhang, T.-H.; Zhan, R.; Li, X.; Li, J.-F.; Tian, J.-H.; Yang, R. Nico Alloy Nanoparticles Decorated on N-Doped Carbon Nanofibers as Highly Active and Durable Oxygen Electrocatalyst. *Advanced Functional Materials* **2018**, *28*, 1705094.

(11) Yan, J.; Wang, Y.; Zhang, Y.; Xia, S.; Yu, J.; Ding, B. Direct Magnetic Reinforcement of Electrocatalytic Orr/Oer with Electromagnetic Induction of Magnetic Catalysts. *Advanced Materials* **2021**, *33*, 2007525.

(12) Li, C.; Wu, M.; Liu, R. High-Performance Bifunctional Oxygen Electrocatalysts for Zinc-Air Batteries over Mesoporous Fe/Co-N-C Nanofibers with Embedding Feco Alloy Nanoparticles. *Applied Catalysis B: Environmental* **2019**, *244*, 150-158.
